# Dissociable contribution of the parietal and frontal cortex to coding movement direction and amplitude

**DOI:** 10.3389/fnhum.2015.00241

**Published:** 2015-05-06

**Authors:** Marco Davare, Alexandre Zénon, Michel Desmurget, Etienne Olivier

**Affiliations:** ^1^Institute of Neuroscience (IoNS), School of Medicine, University of LouvainBrussels, Belgium; ^2^Sobell Department of Motor Neuroscience and Movement Disorders, UCL Institute of Neurology, University College LondonLondon, UK; ^3^Department of Kinesiology, Movement Control and Neuroplasticity Research Group, Biomedical Sciences Group, KU LeuvenLeuven, Belgium; ^4^Centre de Neurosciences Cognitives, CNRS, UMR 5229Bron, France

**Keywords:** action planning, prehension, goal-directed, step-tracking, sensorimotor transformation, posterior parietal cortex, reaching, transcranial magnetic stimulation

## Abstract

To reach for an object, we must convert its spatial location into an appropriate motor command, merging movement direction and amplitude. In humans, it has been suggested that this visuo-motor transformation occurs in a dorsomedial parieto-frontal pathway, although the causal contribution of the areas constituting the “reaching circuit” remains unknown. Here we used transcranial magnetic stimulation (TMS) in healthy volunteers to disrupt the function of either the medial intraparietal area (mIPS) or dorsal premotor cortex (PMd), in each hemisphere. The task consisted in performing step-tracking movements with the right wrist towards targets located in different directions and eccentricities; targets were either visible for the whole trial (Target-ON) or flashed for 200 ms (Target-OFF). Left and right mIPS disruption led to errors in the initial direction of movements performed towards contralateral targets. These errors were corrected online in the Target-ON condition but when the target was flashed for 200 ms, mIPS TMS manifested as a larger endpoint spreading. In contrast, left PMd virtual lesions led to higher acceleration and velocity peaks—two parameters typically used to probe the planned movement amplitude—irrespective of the target position, hemifield and presentation condition; in the Target-OFF condition, left PMd TMS induced overshooting and increased the endpoint dispersion along the axis of the target direction. These results indicate that left PMd intervenes in coding amplitude during movement preparation. The critical TMS timings leading to errors in direction and amplitude were different, namely 160–100 ms before movement onset for mIPS and 100–40 ms for left PMd. TMS applied over right PMd had no significant effect. These results demonstrate that, during motor preparation, direction and amplitude of goal-directed movements are processed by different cortical areas, at distinct timings, and according to a specific hemispheric organization.

## Introduction

Visually-guided movements require sensory information about the target to be extracted and transformed into an appropriate motor command (Crawford et al., 2011; Vesia and Crawford, 2012). In the particular instance of arm movements aimed at grabbing an object, two types of visual information related to object’s extrinsic and intrinsic features need to be extracted to feed two independent transformation processes, which lead to two separate movement components i.e., a reaching (transport of the hand) and a grasping (pre-shaping of the hand posture) component (Jeannerod et al., 1995; Jeannerod, 1997). A large number of experiments in both human and non-human primates corroborated the view that two separate pathways, connecting the posterior parietal cortex (PPC) to the premotor cortex, subserve the two movement components underlying prehension, allowing primates to interact so skillfully with their environment (Jeannerod et al., 1995; Castiello, 2005; Culham et al., 2006). According to this classical view, the reaching component is subserved by a dorsomedial pathway, connecting the medial part of IPS (mIPS) and parieto-occipital junction (POJ) to the dorsal premotor cortex (PMd) and the grasping component relies on a dorsolateral circuit connecting the anterior intraparietal (AIP) and ventral premotor (PMv) cortex (Jeannerod et al., 1995; Castiello, 2005; Davare et al., 2011) although some findings challenge the view that these two circuits process reach and grasp independently (Raos et al., 2004; Fattori et al., 2012). A comparable organization has been evidenced in non-human primates although homologies between human and monkey PPC remain debated (Mars et al., 2011; Vesia and Crawford, 2012; Andersen et al., 2014; Turella and Lingnau, 2014).

However, the question of the causal contribution of individual areas belonging to this “reaching pathway” remains open. In humans, the technique of choice to address this issue is transcranial magnetic stimulation (TMS), and like pharmacological inactivation in monkeys, this approach has the advantage of having a relatively good spatial resolution and of precluding long-term compensation, which might hamper the conclusions of clinical studies. Additionally, TMS offers the unique possibility of establishing the time course of the contribution of the studied area to the task at hand, a piece of information normally unavailable from functional imaging studies (but see Gallivan et al., 2011a,b). We have already used TMS to investigate the grasping circuit in details, deciphering both the causal role of the anterior part of the intraparietal sulcus (aIPS) and PMv, and the time course of their respective contribution when planning a grasping movement (Davare et al., 2006, 2007a; Olivier et al., 2007). More recently we applied the same approach to mIPS, one of the key nodes of the reaching circuit, and found that, during motor preparation, this area encodes the direction of goal-directed movements performed towards contralateral targets (Davare et al., 2012).

However, in addition to movement direction, planning a reaching movement also requires specifying its amplitude. Interestingly during the last two decades, many behavioral studies have suggested that movement direction and amplitude are processed separately, by showing, for instance, that the variability and systematic biases of direction and amplitude errors are independent (Gordon et al., 1994; Messier and Kalaska, 1997, 1999; Vindras et al., 2005). However, whereas, in both humans and monkeys, the direction coding in the primary motor cortex (M1), premotor areas, and the PPC has been well documented (Caminiti et al., 1991; Georgopoulos, 1995; Kakei et al., 1999, 2001; Eisenberg et al., 2010; Fabbri et al., 2010), the neural correlates of amplitude coding are less clear (Riehle and Requin, 1989; Kurata, 1993; Riehle et al., 1994; Fu et al., 1995; Desmurget et al., 1998; Messier and Kalaska, 2000). In monkeys, it has been shown that most cells in PMd encode both movement direction and amplitude (Kurata, 1993), and that this coding possibly occurs serially at a single-cell level, with direction coding occurring first during movement preparation, followed by amplitude coding, spreading over movement execution (Fu et al., 1995). More recently, it has been confirmed that cells coding only for movement amplitude are very uncommon in monkey PMd and that most cells encode both direction and amplitude at some points during the performance of an instructed-delay reaching task (Messier and Kalaska, 2000). Similarly in the parietal cortex, recent studies have also found cells encoding amplitude when monkeys performed reaching movements in 3D requiring different depths (Bhattacharyya et al., 2009). Again, direction and amplitude jointly influenced cell-spiking activity in the earlier planning stages of the reach, while amplitude coding became stronger towards movement execution (Hadjidimitrakis et al., 2014). In humans, sensitivity to movement direction has been demonstrated in a large number of cortical areas including M1, PMd and the parietal reach region (PRR; Eisenberg et al., 2010; Fabbri et al., 2010, 2014), with the strongest directional selectivity in the right PRR, then decreasing in the frontal areas (Fabbri et al., 2010). In an attempt to identify cortical areas involved in amplitude coding, Fabbri and collaborators also investigated the sensitivity to movement amplitude in those parietal and frontal regions known to be tuned to movement direction (Fabbri et al., 2012). This study demonstrated that all PPC regions showing directional tuning for reaching movements (inferior parietal lobule (IPL), aIPS, posterior intraparietal sulcus (pIPS) and superior parieto-occipital cortex (SPOC)) are also sensitive to movement amplitude, in contrast with the conclusion of a TMS study showing no evidence for amplitude coding in mIPS (Davare et al., 2012). In addition, the authors reported that the frontal areas, including PMd, show only a partial transfer from the adapted to the non-adapted amplitude, suggesting that the amplitude might be processed differently in parietal and frontal areas (Fabbri et al., 2012). However, these approaches are correlative and a covariation of neural activity with the movement direction and/or amplitude does not prove that the area under investigation is causally involved in processing these parameters (Messier and Kalaska, 2000).

In order to establish the causal contribution of the dorsomedial pathway to the movement amplitude and/or direction coding in humans, we used TMS to interfere with the function of two of the key nodes belonging to the “reaching” circuit, namely mIPS and PMd, on both sides. TMS was applied in healthy volunteers while performing goal-directed wrist movements with a manipulandum (Hoffman and Strick, 1986) operated with the right hand. The movements were executed towards visual targets located at different direction and amplitude in both visual hemifields; the targets either remained visible for the whole movement duration (Target-ON) or were flashed for 200 ms before movement onset (Target-OFF). The latter condition was chosen to further validate our conclusion concerning the causal role of mIPS in coding movement direction, since in our previous study, movements were performed under constant visual feedback (Davare et al., 2012). As far as PMd is concerned, we reasoned that if, as suggested in monkeys, this area encodes both movement direction and amplitude, interfering with the functioning of this area should alter these two movement parameters, possibly at different delays during movement preparation or execution. TMS has already been shown to allow the identification of two different, temporally dissociated, movement parameters within the same cortical area (Davare et al., 2007a).

## Materials and Methods

### Subjects

Six healthy right-handed (Oldfield, 1971) subjects (range 23–29 years) with normal, or corrected to normal, vision gave their informed consent to participate in the present study. None had history of neurological disease. Potential risks of adverse reactions to TMS were evaluated by means of the TMS Adult Safety Screen questionnaire (Keel et al., 2001). The present experiment was approved by the local ethical committee of the Université catholique de Louvain.

### Experimental Setup

Subjects sat comfortably in front of a 19 inches computer screen located at a distance of 60 cm. Their right forearm was fastened midway between pronation and supination while the right hand grasped the handle of a two-axis manipulandum (Hoffman and Strick, 1986; Davare et al., 2007b, 2012). A potentiometer placed on each axis of the manipulandum allowed us to measure wrist displacements in the horizontal (flexion-extension (FE)) and vertical (radial-ulnar (RU)) planes. A yellow cursor (0.4° wide dot) displayed on the screen continuously indicated the manipulandum position. Eye position was monitored by means of an infrared camera (Thomas Recording GmbH) with a 4 ms temporal resolution. Trials were interrupted whenever a saccade occurred during the fixation period (frequency: 4.1 ± 1.2%, mean ± SD, *n* = 6). Those aborted trials were then replayed at the end of the experiment until all trials had been executed correctly.

### Transcranial Magnetic Stimulation

Single-pulse TMS was delivered through a 70 mm figure-of-eight coil connected to a Magstim 200 stimulator (Magstim, Whitland, UK). Before each experiment, the resting motor threshold—defined as the intensity for which single-pulse TMS applied over the primary motor cortex produced a wrist movement in 50% of the cases—was estimated while the subjects were comfortably seated with their hand relaxed on their lap. TMS intensity was then set at 120% of the resting motor threshold for the whole experimental session. Trials with and without TMS were randomly intermixed during each experimental block.

**Figure 1:**
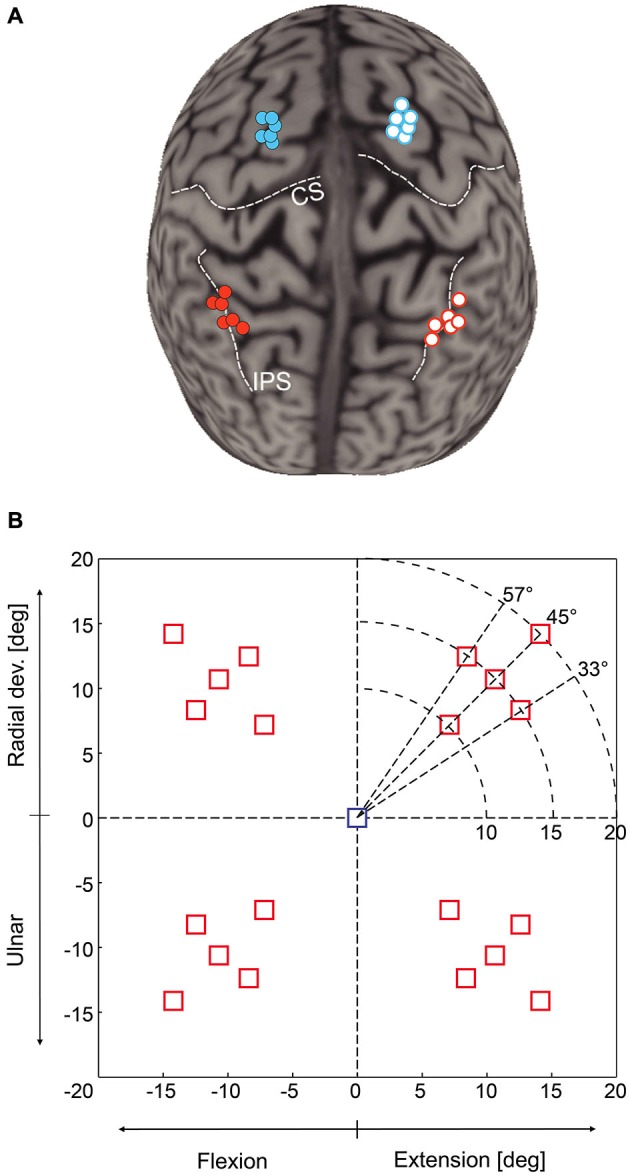
**(A)** Location of transcranial magnetic stimulation (TMS) sites over mIPS and PMd in the left and right hemispheres after normalization into the MNI coordinate system (*n* = 6 for each site). The template used for rendering the cortical surface is Colin27 and each normalized stimulation point has been projected on the template cortical surface using the Brainsight stick tool (Rogue research, Montreal). **(B)** Location of the 20 possible target locations on the computer screen; only one target was randomly displayed during a given trial. The central square represents the starting point. The horizontal axis is the flexion-extension axis of the wrist, the vertical one the radial-ulnar deviation axis. In each quadrant, there were 5 possible target locations: 3 directions (45 ± 12°) and for the target along the 45° axis, 3 possible eccentricities (10, 15 or 20°). Targets were either displayed for the whole movement duration (Target-ON condition) or flashed only for 200 ms (Target-OFF condition).

We used a neuronavigation technique (Noirhomme et al., 2004; Zosso et al., 2006) to place the TMS coil either over mIPS or PMd in either the left or right hemisphere, as identified in the literature (Connolly et al., 2003; Prado et al., 2005; Davare et al., 2012). Anatomical landmarks were used to guide coil placement: PMd was located as the most caudal portion of the superior frontal gyrus at the level of its intersection with the precentral gyrus; mIPS was located over the medial portion of the IPS, near the caudal part of the angular gyrus (see Davare et al., 2012). The mean normalized MNI coordinates of our stimulations points were −33, −47, 48 and 31, −45, 53 mm for left and right mIPS, respectively, and −21, −6, 68 and 22, −7, 65 for left and right PMd, respectively (x, y, z, *n* = 6) (Figure 1A).

### Experimental Procedure

Each trial started with the wrist in a neutral position, a condition fulfilled when the cursor indicating the position of the manipulandum (yellow circle) was at the center of the screen, indicated by a 15 mm (1.5° of field of view) blue square (Figure 1B). Subjects were instructed to fixate this central cue at the beginning of each trial. After a 700 ms delay, the central cue was turned off, replaced by a fixation cross, and a 15 mm red square target was randomly displayed in one out of the 20 possible locations. There were 5 possible target locations in each quadrant, corresponding either to a 45° direction and to a wrist movement amplitude of 10, 15 or 20°, or to a fixed 15° movement amplitude in a 33, 45 or 57° direction (see Figure 1B). We also varied the duration of visual feedback to disentangle online corrective mechanisms from movement planning processes. Targets were displayed either briefly, for 200 ms (Target-OFF condition) or during the whole trial duration (Target-ON condition). Subjects were instructed to move the cursor into the target as rapidly and as accurately as possible. In the Target-ON condition, subjects had to keep the cursor inside the target for at least 700 ms to complete the trial. In the Target-OFF condition, the end of the trial occurred when the cursor velocity dropped below 5% of the velocity peak and remained stable for at least 700 ms. Inter-trial interval varied randomly from 3.5 to 5 s. Throughout the trial, subjects had to maintain eye fixation on the central cross in order to prevent any confounding effects of TMS on eye movements, which could have had indirect consequences on the planning of hand reaching movements.

The experiment consisted of eight blocks of 200 trials in which TMS was applied either over the left or right mIPS or PMd (in separate blocks, 2 blocks each). The order of the 8 blocks was pseudorandomly distributed across subjects. In 80% of the cases, single pulse TMS was delivered either at 100 or 200 ms after target presentation and, in 20% of the cases, no TMS pulse was delivered but the coil remained in place over the current stimulation site.

### Data Acquisition and Analysis

The position signal from the 2 potentiometers was digitized (sampling rate: 1 kHz; PCI-6023E, National Instruments, Austin, TX), and stored on a personal computer for offline analysis. Then, these signals were low-pass filtered offline (16 Hz) with a fourth order, zero-phase-lag, Butterworth filter (see Davare et al., 2007b for details).

We measured the following movement variables: (1) the reaction time (RT), defined as the delay between target onset and the moment when wrist position first exceeded the mean baseline position by 2 SD or more; (2) The movement time (MT), defined as the delay between wrist movement onset and the time the cursor velocity dropped below 5% of the velocity peak and remained stable for at least 700 ms in the Target-OFF condition or inside the target for the Target-ON condition; (3) The displacement ratio (DR), measured by computing the ratio between the total distance traveled by the wrist and the distance between the start and end point of the trajectory. DR provides a reliable estimate of the length of movement trajectory; a DR value equal to 1 corresponds to a straight wrist displacement from the screen center to the target (Davare et al., 2007b, 2012); (4) The value of acceleration and velocity peaks, both known to be linearly related to movement amplitude (Hoffman and Strick, 1986); (5, 6) The mean value and standard deviation (SD) of the initial movement direction were computed to estimate, respectively, the constant (DIR_CE_) and variable direction errors (DIR_VE_). The initial movement direction was measured by computing the direction of the velocity vector at the peak acceleration. This parameter allowed us to determine the direction of the movement initially planned, before any visual feedback may take place (Prablanc and Martin, 1992; Desmurget et al., 2005), as indicated by the early occurrence of the peak acceleration (28.7 ± 8 ms in the present study, mean ± SD). DIR_CE_ was computed by taking the difference between the initial movement direction and the direction of the target; (7) Endpoints of step-tracking movements were only computed for the Target-OFF condition. They were measured at the end of the movement and segregated into endpoint constant and variable errors (Desmurget et al., 1999). Endpoint constant errors were computed as the Euclidean distance between the target and endpoint locations. Endpoint variable errors were estimated by measuring the area of the isodensity ellipsoid in which 95% of the endpoints were located; and (8) The shape of the isodensity ellipsoid was determined by computing the ratio between the length of the long and short axes. In order to group the endpoints for the 10 target locations presented in each hemifield, endpoint coordinates were normalized to the 15° target amplitude and 45° target direction (see Figure 1B) by rotating and scaling the horizontal and vertical coordinates of the endpoints.

**Figure 2:**
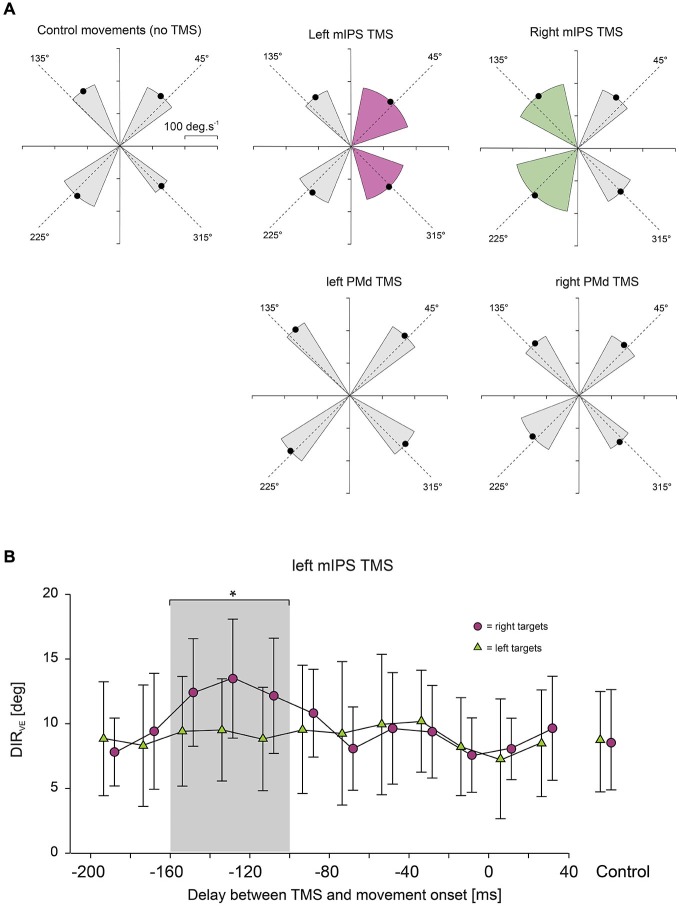
**(A)** Effect of mIPS and PMd TMS on the initial movement direction. Polar plots showing the direction and amplitude of the velocity vector computed at time of peak acceleration. *X*- and *Y*-axes are expressed in deg.s^−1^. For the sake of clarity, only the 4 main target directions are illustrated. The dashed lines represent the actual target directions and the four black dots indicate the mean direction and amplitude (*n* = 6) of the velocity vector for the movements directed to each quadrant; the gray sectors indicate ±2 SD in the control condition or when no TMS effect was revealed. Only TMS applied over mIPS increased the variability in the initial movement direction, and only for movements directed towards contralateral targets, as shown in purple for left mIPS and in green for right mIPS (averages were only computed for the specific TMS timing showing a significant effect, namely 160–100 ms). PMd TMS had no effect on the initial movement direction when compared to control (no TMS). **(B)** Time course of the effects of left mIPS TMS on the initial movement direction (DIR_VE_). Data were assigned to bins of 20 ms width (see Methods). *X*-axis: delay between TMS and movement onset. *Y*-axis: variable error in the initial movement direction expressed in degrees. TMS over left mIPS increased DIR_VE_ only when applied in a given time window during movement preparation, namely 160–100 ms before movement onset; the gray shading area highlights data points significantly different from control (no TMS) conditions, shown in the right-hand side of the graph. Error bars illustrate ±1 SD. Results for right mIPS TMS were the same, but only for left targets (not shown).

**Figure 3:**
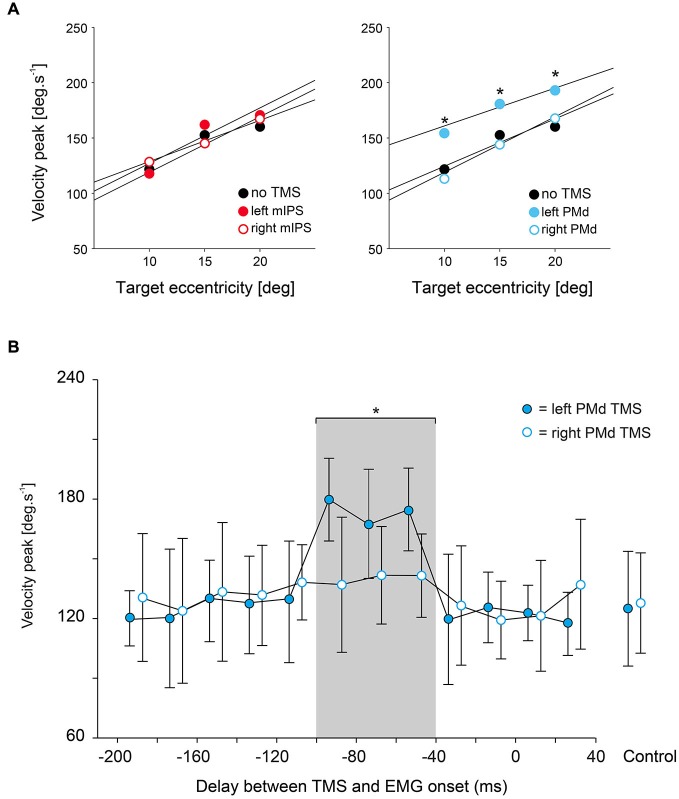
**Effect of mIPS and PMd TMS on peak velocity. (A)** Average peak velocity values (*n* = 6) are shown for the 3 target eccentricities (10, 15 and 20°) and for mIPS (left) and PMd (right) conditions. Values from all target directions and hemifield were pooled together. For the mIPS and PMd TMS conditions, averages were only computed for the specific TMS timings showing a significant effect, namely 160–100 ms and 100–40 ms, respectively. Only TMS applied over left PMd affected right hand movement velocity. Similar effects were found for acceleration peak values (not shown). **(B)** Time course of the effects of left PMd virtual lesions. Data were assigned to bins of 20 ms width. *X*-axis: delay between TMS triggering and movement onset. *Y*-axis: peak velocity. TMS over left PMd increased velocity peak only when applied in 100–40 ms before movement onset; the gray shading area highlights data points significantly different from control (no TMS) condition. Error bars illustrate ±1 SD. TMS applied over right PMd had not affect on velocity peaks of movements performed with the right hand.

**Figure 4:**
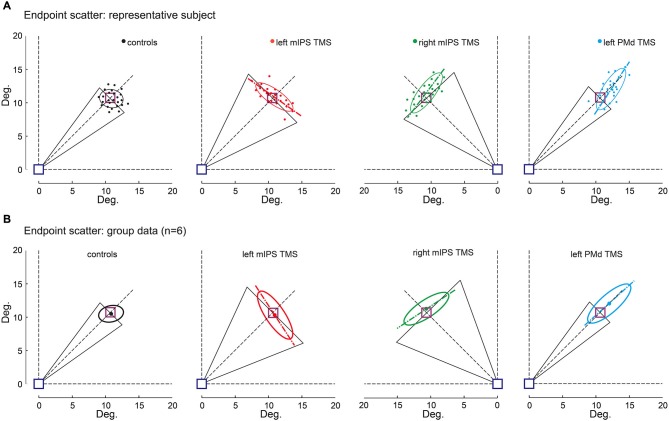
**Effect of TMS on movement endpoint scattering**. Movement endpoints gathered in the OFF-target condition are shown for control (no-TMS), left and right mIPS and left PMd TMS conditions, and both for a representative participant **(A)** and for the group **(B)**. The blue square indicates the starting point and the purple one the normalized target location (see Methods). Note that for mIPS and PMd TMS conditions, averages were computed only for data gathered in the specific TMS time window, namely 160–100 ms for left and right mIPS and 100–40 ms for left PMd. The ellipses illustrate the 95% confidence interval of endpoint locations. The long axis of ellipses is shown to highlight the direction of the main component of endpoint variability. The black triangles represent DIR_VE_ (as in Figure 2), extrapolated up to the target location (purple square). This figure clearly illustrates the distinct effects of TMS applied over mIPS and PMd on movement direction and amplitude, respectively. Left and right mIPS TMS increased the endpoint scattering along a direction orthogonal to the optimal movement path (45° dotted lines) for targets located in the contralateral hemifield, reminiscent of an error in computing the appropriate movement direction. TMS applied over the left PMd led to an overshoot and increased the spread of endpoints along the optimal movement path to reach the targets, irrespective of their location in the right or left hemifield; this effect is illustrative of an inappropriate amplitude programing. **(A)** Representative subject, each dot represents an individual trial. **(B)** Average ellipses across all subjects (*n* = 6). The dot represents the center of each average ellipse.

In order to determine more precisely the time course of the effects of TMS applied over mIPS and PMd on those different movement parameters, each trial was classified with respect to the actual delay between TMS and movement onset and was assigned to a 20 ms time bin (12 bins, ranging from 200 ms before movement onset to 40 ms after movement onset). For each subject and for each bin, an averaged value of the studied parameter was computed, provided that at least 3 data points were available; mean values in each bin were then averaged for all subjects (Davare et al., 2007b). Figures 2–4 only show data for the specific time window TMS was effective (i.e., 100–160 ms bins for mIPS and 40–100 ms bins for PMd.

### Statistical Analysis

First, we performed repeated-measure analyses of variance (ANOVA-RM) for each TMS site separately (left mIPS, right mIPS, left PMd and right PMd), with TMS DELAY (13 levels: no-TMS and 12 bins), HEMIFIELD (ipsi- or contralateral) and target DISPLAY conditions (Target-ON or Target-OFF) as within-subject factors. In order to increase the number of data points per condition and per subject when analysing the movement variables, we pooled together all targets of different eccentricities and directions from the same hemifield. All movement parameters were averaged per condition within each subject. The ANOVA-RM analyses were performed with the SAS Enterprise Guide software, Version 5.1. (Copyright © 2012 SAS Institute Inc., Cary, NC, USA). We performed planned comparisons, investigating selectively the relevant contrasts, and used Tukey correction for multiple comparisons. Then, for each movement parameter, we performed a global analysis including data from all TMS SITES, including them as additional factor.

We analyzed the effects of target eccentricity (10, 15 or 20°) on the peaks of movement derivatives (velocity and acceleration) by means of multiple linear regressions including subjects dummy variables to take into account the correlation between repeated measures. For this analysis, we pooled all target directions together so as to find the global correlation between these movement derivatives and target eccentricity.

## Results

### Effects of TMS on Initial Movement Direction

TMS applied over the left or right mIPS, but not the left or right PMd, altered MT, DR and DIR_VE_, in agreement with our previous findings (Davare et al., 2012). For these parameters, we found a significant DELAY × HEMIFIELD interaction for both mIPS sites (all *F*_(12,260)_ > 3.80, all *p* < 0.0001 for left mIPS TMS; all *F*_(12,260)_ > 2.20, all *p* < 0.012 for right mIPS TMS). Planned comparisons of all bins against the no-TMS condition, for each hemifield, revealed that, TMS applied over the left mIPS, led to an increase in MT, DR and DIR_VE_ only for movements directed towards contralateral targets (Figure 2A), and only when TMS was delivered 160–100 ms before movement onset (all *t*_(260)_ > 3.06, all *p* < 0.0025; Figure 2B). Identical results were found following right mIPS TMS: MT, DR and DIR_VE_ increased only for movements performed towards contralateral targets (Figure 2A) when TMS was applied 160–100 ms before movement onset (all *t*_(260)_ > 2.54, all *p* < 0.012). There was no significant main effect of DISPLAY (Target-ON vs. Target-OFF condition) nor DELAY × DISPLAY effect on these movement parameters (DISPLAY: all *F*_(1,260)_ < 2.0, all *p* > 0.15; DELAY × DISPLAY: all *F*_(12,260)_ < 1.6, all *p* > 0.1). Left or right PMd virtual lesions did not affect MT, DR and DIR_VE_ (Figure 2A), as shown by an absence of significant main effect of DELAY or interaction between DELAY and HEMIFIELD on these parameters (all *p* > 0.2).

A global analysis including all TMS sites confirmed a significant SITE × HEMIFIELD × DELAY interaction (*F*_(12,520)_ = 2.56, *p* = 0.0027), with a planned comparison confirming that the difference between the no-TMS condition and the 160–100 ms bins was significantly higher for both mIPS sites than for both PMd sites (*t*_(520)_ = 4.03, *p* < 0.0001).

### Effects of TMS on Movement Acceleration and Velocity

We found that only TMS applied over left PMd affected the acceleration and velocity peaks; TMS applied over right PMd and over either mIPS had no effect on these parameters. ANOVA-RM showed a significant main effect of DELAY for the acceleration and velocity peaks only when TMS was applied over left PMd (*F*_(12,260)_ = 9.00, *p* < 0.001 and *F*_(12,260)_ = 28.99, *p* < 0.0001, respectively; Figure 3A). Planned comparisons revealed that applying TMS over the left PMd 100–40 ms before movement onset led to an overall increase in the acceleration and velocity peaks (all *t*_(260)_ > 3, all *p* < 0.0027, Figure 3B); this effect was observed for all target locations (main effect of HEMIFIELD and DELAY × HEMIFIELD interaction: all *p* > 0.2). TMS applied over left or right mIPS did not affect the peak of velocity or acceleration as denoted by a lack of main effect of DELAY and of interaction between DELAY and HEMIFIELD (all *p* > 0.18) on these parameters. A global analysis on all TMS sites confirmed a significant SITE × DELAY interaction for both velocity and acceleration peaks (*F*_(36,1040)_ > 3.29, *p* < 0.0001), and the planned comparison showed that the difference between the no-TMS condition and the bins between 100 and 40 ms was significantly larger for left PMd than for all other stimulation sites (*t*_(1144)_ = 2, *p* = 0.0455). There was no main effect of DISPLAY (Target-ON vs. Target-OFF condition) on acceleration and velocity peaks (*F*_(1,260)_ < 2.0, *p* > 0.16).

In the no-TMS condition, linear regressions between acceleration or velocity peaks and target eccentricity showed the typical relationship between the peaks of movement derivatives and movement amplitude (see Figure 3A; Acceleration: Intercept = 0.83 × 10^3^, Slope = 5.32, *p* < 0.001; Velocity: Intercept = 105.15, Slope = 4.304, *p* < 0.001). When TMS was applied over the left PMd 100–40 ms before movement onset, the intercept of the linear regression increased significantly (acceleration: Intercept = 1.34 × 10^3^, *p* < 0.001; velocity: Intercept = 147.28, *p* < 0.001, Figure 3A), mainly due to the increased peak of movement derivatives after left PMd virtual lesions. This effect was also found irrespective of target location (HEMIFIELD main effect or SITE × HEMIFIELD interaction: all *F* < 1) and was comparable for both target display conditions ( DISPLAY main effect or SITE × DISPLAY interaction: all *F* < 1). In contrast, the slope of the regression line relationship between peak derivatives and target eccentricity was not significantly affected (Acceleration: Slope = 5.902; Velocity: Slope = 4.523, all *p* > 0.05; Figure 3A).

### Effects of TMS on Movement Endpoints

The Target-OFF condition allowed us to separate the effect of TMS on movement planning from the online corrective mechanisms (Prablanc et al., 2003). Indeed in the target-OFF condition, hand movements started well after the target was switched off, which prevented the hand trajectory to be updated based on visual feedback about target location, thus mostly relying on the initial movement plan. The endpoint constant error increased following left PMd virtual lesions only when TMS was delivered 100–40 ms before movement onset (main effect of DELAY : *F*_(12,130)_ = 21, *p* < 0.0001, planned comparisons: all *t*_(130)_ > 1.86, *p* < 0.0001), irrespective of target location ( HEMIFIELD main effect and DELAY × HEMIFIELD interaction, both *F* < 1.2, *p* > 0.3). Figure 4 shows that following left PMd virtual lesions the step-tracking movements systematically overshot the target whereas the endpoint constant error was not affected by TMS applied over the right PMd nor over left mIPS or right mIPS (not shown). A global ANOVA-RM performed on all TMS sites showed a significant SITE × DELAY interaction (*F*_(36,520)_ = 6.84, *p* < 0.0001). A planned comparison confirmed that the difference between the no-TMS condition and the 100–40 ms bins was significantly larger for left PMd than for the other stimulation sites (*t*_(1144)_ = 4.85, *p* < 0.0001).

The endpoint variable error was affected when TMS was applied over left mIPS, right mIPS (not shown) or left PMd (all *F*_(12,130)_ > 28, *p* < 0.0001; see Figure 4). Following left or right mIPS TMS, the endpoint variable errors increased when TMS was applied 160–100 ms before movement onset and for movements directed towards contralateral targets (DELAY × HEMIFIELD interaction: *F*_(12,130)_ > 29, both *p* < 0.0001, all planned comparisons, *t*_(130)_ > 10, *p* < 0.0001). Importantly, the shape of the isodensity ellipsoid (ratio between the lengths of long and short axes) was different for both left and right mIPS TMS conditions when compared to controls (both *t*_(5)_ > 5.24, all *p* < 0.025). For both mIPS TMS conditions, the axis that explains the largest endpoint variability was nearly orthogonal (85 ± 17°, mean ± SD, *n* = 6) to the optimal movement direction (Figure 4). This peculiar ellipsoid shape was found only when TMS was delivered 160–100 ms before the onset of movements directed towards contralateral targets (all *p* < 0.043).

For left PMd virtual lesions, the endpoint variable error increased only when TMS was applied 100–40 ms before movement onset and for movements towards all targets (*F*_(12,130)_ = 55.64, *p* < 0.0001; planned comparisons: all *t*_(130)_ > 10, all *p* < 0.0001). In contrast to results gathered for mIPS, TMS applied over left PMd led to ellipsoid shapes that extended along the target direction (all *t*_(5)_ > 6.76, all *p* < 0.004), with their long axis roughly aligned with the optimal movement path to reach the target (Figure 4, 3 ± 12°, mean ± SD, *n* = 6). Finally, a global ANOVA-RM on endpoint variable error confirmed a significant SITE × HEMIFIELD × DELAY effect (*F*_(36,520)_ = 17.01, *p* < 0.0001). We performed a planned comparison highlighting the specific mIPS effect, comparing the no-TMS vs. 160–100 ms bins difference between mIPS and PMd (*t*_(520)_ = 3.09, *p* = 0.0021) and another comparison focused on the specific effect obtained on left PMD (no-TMS vs. 100–40 ms bins, left PMD vs. other areas: *t*_(520)_ = 10.19, *p* < 0.0001).

## Discussion

The present study indicates that direction and amplitude coding of goal-directed movements is performed by two distinct areas belonging to the dorsomedial “reaching” pathway, with direction being processed, first, in the posterior parietal cortex (mIPS) and amplitude being implemented later in the premotor cortex (PMd). In addition, we found that both mIPS are involved in coding the direction of right hand movements when performed in the contralateral hemifield, whereas left PMd processes the amplitude of all right hand displacements, whatever the location of the target, in the right or left hemifield. These results shed new light onto the cascade of visuo-motor transformations performed in the dorsomedial pathway of the prehension circuit.

The finding that both mIPS process the direction of goal-directed movements performed towards contralateral targets during movement preparation corroborates and extends our previous study (Davare et al., 2012), by showing that, when no visual feed-back was available during the task performance (Target-OFF condition), the movement endpoint distribution was biased along a direction orthogonal to the optimal movement path, and again only for movements directed towards contralateral targets and within a precise time window (160–100 ms) before movement onset. In our previous study, a series of control studies allowed us to conclude that the effect of mIPS TMS on movement direction could not be explained by an incorrect target spatial representation but was due to an inaccurate computation of the reach “motor” vector, or more precisely of the direction of this vector since our previous, and current, results have failed to reveal any evidence that mIPS is involved in coding the norm (i.e., amplitude) of this movement vector (Davare et al., 2012). Importantly, because we were able to demonstrate a deficit in coding the movement amplitude when TMS was applied over PMd (see below), the negative results we obtained following mIPS TMS do not results from a lack of accuracy or sensitivity of the measurements and/or analyses we performed.

As previously mentioned in the Introduction, the contribution of the PPC to movement direction coding has already been suggested in humans, in particular by using fMRI adaptation protocols (Fabbri et al., 2010). In this study, the same movement was repeated several times in one particular direction, followed by a test trial consisting of a movement executed in a different direction. The rationale of this approach is that, if the investigated area contains a directionally tuned cell population, its activation during the test trial should be proportional to the angular difference between the adapted and tested directions (Fabbri et al., 2010). Such an approach has allowed the authors to identify an extensive network of cortical areas sensitive to the direction of reaching movements, namely bilateral PMd, mIPS, aIPS and PRR (Fabbri et al., 2010), but also SMA and anterior precuneus (Fabbri et al., 2012); additionally, an interaction between reach direction and grip type has been found in a large number of areas belonging to the dorsolateral “grasping” circuit, including PMv (Fabbri et al., 2014). The large number of areas that this technique has revealed as being directionally sensitive, together with the fact that eye movements and/or attention allocation might have influenced these results, raises the issue of the causal role of all these areas in movement direction processing. Another possible drawback of these studies is that they did not try to disentangle movement direction from the other parameters that systematically covary with it, such as kinematics, EMG activity, or pattern of joint rotations. In any case, a modulation of the BOLD signal with movement direction alone cannot be viewed as evidence for a causal role in coding this parameter, as proved by the discrepancy between, for example, the lack of evidence for direction coding in PMd (current study) and the results of these fMRI adaptation studies, all showing consistently that this area contains directionally tuned cell populations.

Surprisingly, a lot fewer studies have investigated the role of cortical areas in coding movement amplitude. In monkeys, cells coding for amplitude have been mainly reported in PMd, but it has been repeatedly shown that most PMd cells encode both direction and amplitude of reaching movements (Kurata, 1993), in a sequential order, with movement direction being coded first, then followed by amplitude (Fu et al., 1995). The scarcity (around 2–4%) of cells coding only for movement amplitude in PMd has been confirmed by the group of Kalaska, supporting the view that most PMd cells in monkeys have an activity serially related to both direction and amplitude, during the performance of an instructed-delay reaching task (Messier and Kalaska, 2000). More recently, it has been shown in the monkey that the activity of most cells in V6A is also modulated by both depth and direction during reaching (Hadjidimitrakis et al., 2014), in contrast with the conclusion of the only study showing the existence of distinct cell populations coding the distance, azimuth and elevation in area 5 in monkey PPC (Lacquaniti et al., 1995).

Likewise, in humans, only very few studies have directly explored the coding of movement amplitude, possibly because of the even larger number of movement parameters that covary with amplitude (Messier and Kalaska, 2000). Recently, Fabbri and collaborators investigated the sensitivity to movement amplitude in those parietal and frontal regions already known to be tuned to movement direction (Fabbri et al., 2012). This study demonstrated that all PPC areas directionally tuned for direction (IPL, aIPS, pIPS and SPOC) are also sensitive to movement amplitude, suggesting they contain cell populations tuned to specific combinations of direction and amplitude. In addition these authors reported that the frontal areas, including PMd, PMv and SMA, show a partial transfer of adaptation to movement direction from the large to the small movement amplitude, but not the opposite, suggesting that, although this result remains puzzling, the amplitude is somehow processed by frontal areas, but in a different way than in the PPC areas (Fabbri et al., 2012).

The present study fails to find any evidence that mIPS is causally involved in amplitude coding, although of course we cannot exclude that another parietal area belonging to the “reaching” cortical circuit (Vesia and Crawford, 2012) codes movement amplitude. This finding corroborates the conclusion of our earlier TMS study showing no evidence for amplitude coding in mIPS (Davare et al., 2012). However, because this earlier conclusion was based on indirect evidence gathered by analyzing velocity and acceleration peaks, it remained questionable. To address this issue, in the current study, we tested an additional open-loop condition in which the target was flashed only for 200 ms, allowing us to prove that the distribution of endpoint errors after mIPS TMS was compatible with errors in coding direction, and not compatible with an error in movement amplitude, in contrast to what we found for PMd TMS.

From a more theoretical perspective, the current study re-opens the long-lasting debate about a separate coding for amplitude and direction, a view mainly supported by behavioral studies (Desmurget et al., 1998), but for which neural evidence was still lacking, at least at the cortical level. At a behavioral level, a series of findings concur in suggesting independence of treatment for amplitude and direction: RT is decreased by prior information about either the direction or the distance of the target with respect to the hand (Rosenbaum, 1980; Bock and Arnold, 1992; Desmurget et al., 2004); rotation and gain learning occur at different paces and have different patterns of generalization (Pine et al., 1996; Krakauer et al., 2000; Vindras and Viviani, 2002); specification of movement amplitude and direction follow different time courses (Favilla et al., 1989; Ghez et al., 1997); variability and systematic biases of direction and amplitude errors are independent (Gordon et al., 1994; Messier and Kalaska, 1999; Vindras et al., 2005). The current TMS study demonstrates for the first time that such an independent coding for direction and amplitude exists at the cortical level. This finding echoes the results of previous studies that have linked amplitude coding with the basal ganglia (BG) network (Desmurget et al., 2003, 2004; Desmurget and Turner, 2008). Indeed, BG activity is known to modulate neural response in PMd (Grafton et al., 2006). It is thus tempting to speculate that BG inputs mediate the influence of PMd on movement amplitude (or any covariate of this parameter; e.g., velocity, acceleration, force). This view is compatible with recent evidence that the BG modulates movement performance according to non-motor motivational factors (Mazzoni et al., 2007; Turner and Desmurget, 2010; Baraduc et al., 2013). It is also interesting to note that the BG network has been involved in coding force amplitude during grasping movements, likely via BG connections to the dorsolateral grasping circuit including AIP and PMv (Wasson et al., 2010). This highlights that the BG are a key node for coding the amplitude not only for reaching movements via interactions with the dorsomedial circuit but also for force scaling via interactions with the dorsolateral grasping circuit (see Prodoehl et al., 2009 for review). In addition, to this point, and in agreement with electrophysiological experiments performed in monkeys (Fu et al., 1995; Messier and Kalaska, 2000; Hadjidimitrakis et al., 2014), our results also confirm that the amplitude is processed later than the direction information during reach movement preparation, suggesting that these two parameters are processed, or at least implemented in a serial order in the motor-related cortical areas. We can speculate that a serial encoding of direction and amplitude is likely to reflect a motor control strategy leading to smoother movement generation. First selecting a specific agonist muscle group (to reach a particular direction in space) before determining the exact amount of muscle activity required to reach a given distance seems a more cost-saving strategy. Interestingly, the current study also suggests that the processing of motor intention signals evolves along the parieto-frontal circuit: whereas the left or right mIPS only encodes preparatory signals for movements directed towards targets located in the contralateral hemifield, the coding in PMd appears more closely linked to the effector i.e., the contralateral hand, irrespective of the target location. Nevertheless, an additional study in which the left and right hands are systematically tested will be necessary to substantiate this conclusion.

To summarize, the present study provides, for the first time, evidence for a double-dissociation between direction and amplitude coding of reaching movements within the dorsomedial reaching circuit in humans. It is noteworthy that another candidate area for playing a causal role in encoding amplitude within the human dorsomedial circuit could be the human homolog of V6A (Pitzalis et al., 2013). Since Ciavarro et al. (2013) have found endpoint amplitude errors in reaching movements when TMS was applied over that area. Interestingly this effect seemed related more to a disruption of the visuospatial target representation rather than to the motor representation of target amplitude such as following PMd TMS. Further studies are required to substantiate the existence of a visuomotor gradient of amplitude encoding in parieto-frontal networks. Additional TMS experiments are also needed to investigate the possible interactions between the dorsomedial reaching and dorsolateral grasping circuits because, so far, these two components of prehension movements remain frequently investigated by using separate experimental paradigms.

## Conflict of Interest Statement

The authors declare that the research was conducted in the absence of any commercial or financial relationships that could be construed as a potential conflict of interest.

## References

[B1] Andersen R. A. Andersen K. N. Hwang E. J. Hauschild M. (2014). Optic ataxia: from balint’s syndrome to the parietal reach region. Neuron 81, 967–983. 10.1016/j.neuron.2014.02.025 24607223 PMC4000741

[B2] Baraduc P. Thobois S. Gan J. Broussolle E. Desmurget M. (2013). A common optimization principle for motor execution in healthy subjects and parkinsonian patients. J. Neurosci. 33, 665–677. 10.1523/JNEUROSCI.1482-12.2013 23303945 PMC6704928

[B3] Bhattacharyya R. Musallam S. Andersen R. A. (2009). Parietal reach region encodes reach depth using retinal disparity and vergence angle signals. J. Neurophysiol. 102, 805–816. 10.1152/jn.90359.2008 19439678 PMC2724352

[B4] Bock O. Arnold K. (1992). Motor control prior to movement onset: preparatory mechanisms for pointing at visual targets. Exp. Brain Res. 90, 209–216. 10.1007/bf00229273 1521609

[B5] Caminiti R. Johnson P. B. Gailli C. Ferraina S. Burnod Y. (1991). Making arm movements within different parts of space?: the premotor and motor cortical representation of a coordinate system for reaching to visual targets. J. Neurosci. 11, 1182–1197. 2027042 10.1523/JNEUROSCI.11-05-01182.1991 PMC6575326

[B6] Castiello U. (2005). The neuroscience of grasping. Nat. Rev. Neurosci. 6, 726–736. 10.1038/nrn1744 16100518

[B7] Ciavarro M. Ambrosini E. Tosoni A. Committeri G. Fattori P. Galletti C. (2013). rTMS of medial parieto-occipital cortex interferes with attentional reorienting during attention and reaching tasks. J. Cogn. Neurosci. 25, 1453–1462. 10.1162/jocn_a_00409 23647519

[B8] Connolly J. D. Andersen R. A. Goodale M. A. (2003). FMRI evidence for a ‘parietal reach region’ in the human brain. Exp. Brain Res. 153, 140–145. 10.1007/s00221-003-1587-1 12955383

[B9] Crawford J. D. Henriques D. Y. P. Medendorp W. P. (2011). Three-dimensional transformations for goal-directed action. Annu. Rev. Neurosci. 34, 309–331. 10.1146/annurev-neuro-061010-113749 21456958

[B10] Culham J. C. Cavina-Pratesi C. Singhal A. (2006). The role of parietal cortex in visuomotor control: what have we learned from neuroimaging? Neuropsychologia 44, 2668–2684. 10.1016/j.neuropsychologia.2005.11.003 16337974

[B11] Davare M. Andres M. Clerget E. Thonnard J.-L. Olivier E. (2007a). Temporal dissociation between hand shaping and grip force scaling in the anterior intraparietal area. J. Neurosci. 27, 3974–3980. 10.1523/jneurosci.0426-07.2007 17428971 PMC6672552

[B12] Davare M. Andres M. Cosnard G. Thonnard J.-L. Olivier E. (2006). Dissociating the role of ventral and dorsal premotor cortex in precision grasping. J. Neurosci. 26, 2260–2268. 10.1523/jneurosci.3386-05.2006 16495453 PMC6674806

[B13] Davare M. Duque J. Vandermeeren Y. Thonnard J.-L. Olivier E. (2007b). Role of the ipsilateral primary motor cortex in controlling the timing of hand muscle recruitment. Cereb. Cortex 17, 353–362. 10.1093/cercor/bhj152 16525129

[B14] Davare M. Kraskov A. Rothwell J. C. Lemon R. N. (2011). Interactions between areas of the cortical grasping network. Curr. Opin. Neurobiol. 21, 565–570. 10.1016/j.conb.2011.05.021 21696944 PMC3437559

[B15] Davare M. Zénon A. Pourtois G. Desmurget M. Olivier E. (2012). Role of the medial part of the intraparietal sulcus in implementing movement direction. Cereb. Cortex 22, 1382–1394. 10.1093/cercor/bhr210 21862445

[B16] Desmurget M. Epstein C. M. Turner R. S. Prablanc C. Alexander G. E. Grafton S. T. (1999). Role of the posterior parietal cortex in updating reaching movements to a visual target. Nat. Neurosci. 2, 563–567. 10.1038/9219 10448222

[B17] Desmurget M. Grafton S. T. Vindras P. H. Gréa H. Turner R. S. (2003). Basal ganglia network mediates the control of movement amplitude. Exp. Brain Res. 153, 197–209. 10.1007/s00221-003-1593-3 13680045

[B18] Desmurget M. Grafton S. T. Vindras P. Grea H. Turner R. S. (2004). The basal ganglia network mediates the planning of movement amplitude. Eur. J. Neurosci. 19, 2871–2880. 10.1111/j.0953-816x.2004.03395.x 15147320

[B19] Desmurget M. Pélisson D. Rossetti Y. Prablanc C. (1998). From eye to hand: planning goal-directed movements. Neurosci. Biobehav. Rev. 22, 761–788. 10.1016/s0149-7634(98)00004-9 9809311

[B20] Desmurget M. Turner R. S. (2008). Testing basal ganglia motor functions through reversible inactivations in the posterior internal globus pallidus. J. Neurophysiol. 99, 1057–1076. 10.1152/jn.01010.2007 18077663 PMC2906399

[B21] Desmurget M. Turner R. S. Prablanc C. Russo G. S. Alexander G. E. Grafton S. T. (2005). Updating target location at the end of an orienting saccade affects the characteristics of simple point-to-point movements. J. Exp. Psychol. Hum. Percept Perform. 31, 1510–1536. 10.1037/0096-1523.31.6.1510 16366805

[B22] Eisenberg M. Shmuelof L. Vaadia E. Zohary E. (2010). Functional organization of human motor cortex: directional selectivity for movement. J. Neurosci. 30, 8897–8905. 10.1523/jneurosci.0007-10.2010 20592212 PMC6632899

[B23] Fabbri S. Caramazza A. Lingnau A. (2010). Tuning curves for movement direction in the human visuomotor system. J. Neurosci. 30, 13488–13498. 10.1523/jneurosci.2571-10.2010 20926674 PMC6634734

[B24] Fabbri S. Caramazza A. Lingnau A. (2012). Distributed sensitivity for movement amplitude in directionally tuned neuronal populations. J. Neurophysiol. 107, 1845–1856. 10.1152/jn.00435.2011 22205646

[B25] Fabbri S. Strnad L. Caramazza A. Lingnau A. (2014). Overlapping representations for grip type and reach direction. Neuroimage 94, 138–146. 10.1016/j.neuroimage.2014.03.017 24650596

[B26] Fattori P. Breveglieri R. Raos V. Bosco A. Galletti C. (2012). Vision for action in the macaque medial posterior parietal cortex. J. Neurosci. 32, 3221–3234. 10.1523/jneurosci.5358-11.2012 22378893 PMC6622013

[B27] Favilla M. Hening W. Ghez C. (1989). Trajectory control in targeted force impulses. VI. Independent specification of response amplitude and direction. Exp. Brain Res. 75, 280–294. 10.1007/bf00247934 2721609

[B28] Fu Q. G. Flament D. Coltz J. D. Ebner T. J. (1995). Temporal encoding of movement kinematics in the discharge of primate primary motor and premotor neurons. J. Neurophysiol. 73, 836–854. 7760138 10.1152/jn.1995.73.2.836

[B29] Gallivan J. P. McLean D. A. Smith F. W. Culham J. C. (2011a). Decoding effector-dependent and effector-independent movement intentions from human parieto-frontal brain activity. J. Neurosci. 31, 17149–17168. 10.1523/jneurosci.1058-11.2011 22114283 PMC6623835

[B30] Gallivan J. P. McLean D. A. Valyear K. F. Pettypiece C. E. Culham J. C. (2011b). Decoding action intentions from preparatory brain activity in human parieto-frontal networks. J. Neurosci. 31, 9599–9610. 10.1523/jneurosci.0080-11.2011 21715625 PMC6623162

[B31] Georgopoulos A. P. (1995). Current issues in directional motor control. Trends Neurosci. 18, 506–510. 10.1016/0166-2236(95)92775-l 8592761

[B32] Ghez C. Favilla M. Ghilardi M. F. Gordon J. Bermejo R. Pullman S. (1997). Discrete and continuous planning of hand movements and isometric force trajectories. Exp. Brain Res. 115, 217–233. 10.1007/pl00005692 9224851

[B33] Gordon J. Ghilardi M. F. Ghez C. (1994). Accuracy of planar reaching movements. I. Independence of direction and extent variability. Exp. Brain Res. 99, 97–111. 10.1007/bf00241415 7925800

[B34] Grafton S. T. Turner R. S. Desmurget M. Bakay R. Delong M. Vitek J.. (2006). Normalizing motor-related brain activity: subthalamic nucleus stimulation in parkinson disease. Neurology 66, 1192–1199. 10.1212/01.wnl.0000214237.58321.c3 16636237

[B35] Hadjidimitrakis K. Bertozzi F. Breveglieri R. Bosco A. Galletti C. Fattori P. (2014). Common neural substrate for processing depth and direction signals for reaching in the monkey medial posterior parietal cortex. Cereb. Cortex 24, 1645–1657. 10.1093/cercor/bht021 23382514

[B36] Hoffman D. S. Strick P. L. (1986). Step-tracking movements of the wrist in humans. I. Kinematic analysis. J. Neurosci. 6, 3309–3318. 3772433 10.1523/JNEUROSCI.06-11-03309.1986 PMC6568486

[B37] Jeannerod M. (1997). The Cognitive Neuroscience of Action. Oxford: Wiley-Blackwell.

[B38] Jeannerod M. Arbib M. A. Rizzolatti G. Sakata H. (1995). Grasping objects: the cortical mechanisms of visuomotor transformation. Trends Neurosci. 18, 314–320. 10.1016/0166-2236(95)93921-j 7571012

[B40] Kakei S. Hoffman D. S. Strick P. L. (1999). Muscle and movement representations in the primary motor cortex. Science 285, 2136–2139. 10.1126/science.285.5436.2136 10497133

[B41] Kakei S. Hoffman D. S. Strick P. L. (2001). Direction of action is represented in the ventral premotor cortex. Nat. Neurosci. 4, 1020–1025. 10.1038/nn726 11547338

[B42] Keel J. C. Smith M. J. Wassermann E. M. (2001). A safety screening questionnaire for transcranial magnetic stimulation. Clin. Neurophysiol. 112:720. 10.1016/s1388-2457(00)00518-6 11332408

[B43] Krakauer J. W. Pine Z. M. Ghilardi M. F. Ghez C. (2000). Learning of visuomotor transformations for vectorial planning of reaching trajectories. J. Neurosci. 20, 8916–8924. 11102502 10.1523/JNEUROSCI.20-23-08916.2000 PMC6773094

[B44] Kurata K. (1993). Premotor cortex of monkeys?: set- and movement-related activity reflecting amplitude and direction of wrist movements premotor cortex of monkeys?: set- and movement-related reflecting amplitude and direction of wrist movements. J. Neurophysiol. 69, 187–200. 8433130 10.1152/jn.1993.69.1.187

[B45] Lacquaniti F. Guigon E. Bianchi L. Ferraina S. Caminiti R. (1995). Representing spatial information for limb movement?: role of area 5 in the monkey. Cereb. Cortex 5, 391–409. 10.1093/cercor/5.5.391 8547787

[B46] Mars R. B. Jbabdi S. Sallet J. O’Reilly J. X. Croxson P. L. Olivier E.. (2011). Diffusion-weighted imaging tractography-based parcellation of the human parietal cortex and comparison with human and macaque resting-state functional connectivity. J. Neurosci. 31, 4087–4100. 10.1523/JNEUROSCI.5102-10.2011 21411650 PMC3091022

[B47] Mazzoni P. Hristova A. Krakauer J. W. (2007). Why don’t we move faster? Parkinson’s disease, movement vigor, and implicit motivation. J. Neurosci. 27, 7105–7116. 10.1523/jneurosci.0264-07.2007 17611263 PMC6794577

[B48] Messier J. Kalaska J. F. (1997). Differential effect of task conditions on errors of direction and extent of reaching movements. Exp. Brain Res. 115, 469–478. 10.1007/pl00005716 9262201

[B49] Messier J. Kalaska J. F. (1999). Comparison of variability of initial kinematics and endpoints of reaching movements. Exp. Brain Res. 125, 139–152. 10.1007/s002210050669 10204767

[B50] Messier J. Kalaska J. F. (2000). Covariation of primate dorsal premotor cell activity with direction and amplitude during a memorized-delay reaching task. J. Neurophysiol. 84, 152–165. 10899193 10.1152/jn.2000.84.1.152

[B51] Noirhomme Q. Ferrant M. Vandermeeren Y. Olivier E. Macq B. Cuisenaire O. (2004). Registration and real-time visualization of transcranial magnetic stimulation with 3-D MR images. IEEE Trans. Biomed. Eng. 51, 1994–2005. 10.1109/tbme.2004.834266 15536901

[B52] Oldfield R. C. (1971). The assessment and analysis of handedness: the Edinburgh inventory. Neuropsychologia 9, 97–113. 10.1016/0028-3932(71)90067-4 5146491

[B53] Olivier E. Davare M. Andres M. Fadiga L. (2007). Precision grasping in humans: from motor control to cognition. Curr. Opin. Neurobiol. 17, 644–648. 10.1016/j.conb.2008.01.008 18337084

[B54] Pine Z. M. Krakauer J. W. Gordon J. Ghez C. (1996). Learning of scaling factors and reference axes for reaching movements. Neuroreport 7, 2357–2361. 10.1097/00001756-199610020-00016 8951852

[B55] Pitzalis S. Sereno M. I. Committeri G. Fattori P. Galati G. Tosoni A.. (2013). The human homologue of macaque area V6A. Neuroimage 82, 517–530. 10.1016/j.neuroimage.2013.06.026 23770406 PMC3760586

[B56] Prablanc C. Desmurget M. Gréa H. (2003). Neural control of on-line guidance of hand reaching movements. Prog. Brain Res. 142, 155–170. 10.1016/s0079-6123(03)42012-8 12693260

[B57] Prablanc C. Martin O. (1992). Automatic control during hand reaching at undetected two-dimensional target displacements. J. Neurophysiol. 67, 455–469. 1569469 10.1152/jn.1992.67.2.455

[B58] Prado J. Clavagnier S. Otzenberger H. Scheiber C. Kennedy H. Perenin M. T. (2005). Two cortical systems for reaching in central and peripheral vision. Neuron 48, 849–858. 10.1016/j.neuron.2005.10.010 16337921

[B59] Prodoehl J. Corcos D. M. Vaillancourt D. E. (2009). Basal ganglia mechanisms underlying precision grip force control. Neurosci. Biobehav. Rev. 33, 900–908. 10.1016/j.neubiorev.2009.03.004 19428499 PMC2684813

[B60] Raos V. Umiltá M.-A. Gallese V. Fogassi L. (2004). Functional properties of grasping-related neurons in the dorsal premotor area f2 of the macaque monkey. J. Neurophysiol. 92, 1990–2002. 10.1152/jn.00154.2004 15163668

[B61] Riehle A. MacKay W. A. Requin J. (1994). Are extent and force independent movement parameters? Preparation- and movement-related neuronal activity in the monkey cortex. Exp. Brain Res. 99, 56–74. 10.1007/bf00241412 7925796

[B62] Riehle A. Requin J. (1989). Monkey primary motor and premotor cortex: single-cell activity related to prior information about direction and extent of an intended movement. J. Neurophysiol. 61, 534–549. 2709098 10.1152/jn.1989.61.3.534

[B63] Rosenbaum D. A. (1980). Human movement initiation: specification of arm, direction and extent. J. Exp. Psychol. Gen. 109, 444–474. 10.1037/0096-3445.109.4.444 6449531

[B64] Turella L. Lingnau A. (2014). Neural correlates of grasping. Front. Hum. Neurosci. 8:686. 10.3389/fnhum.2014.00686 25249960 PMC4158794

[B65] Turner R. S. Desmurget M. (2010). Basal ganglia contributions to motor control: a vigorous tutor. Curr. Opin. Neurobiol. 20, 704–716. 10.1016/j.conb.2010.08.022 20850966 PMC3025075

[B66] Vesia M. Crawford J. D. (2012). Specialization of reach function in human posterior parietal cortex. Exp. Brain Res. 221, 1–18. 10.1007/s00221-012-3158-9 22777102

[B67] Vindras P. Desmurget M. Viviani P. (2005). Error parsing in visuomotor pointing reveals independent processing of amplitude and direction. J. Neurophysiol. 94, 1212–1224. 10.1152/jn.01295.2004 15857965

[B68] Vindras P. Viviani P. (2002). Altering the visuomotor gain: evidence that motor plans deal with vector quantities. Exp. Brain Res. 147, 280–295. 10.1007/s00221-002-1211-9 12428136

[B69] Wasson P. Prodoehl J. Coombes S. A. Corcos D. M. Vaillancourt D. E. (2010). Predicting grip force amplitude involves circuits in the anterior basal ganglia. Neuroimage 49, 3230–3238. 10.1016/j.neuroimage.2009.11.047 19944767 PMC2818558

[B70] Zosso D. Noirhomme Q. Davare M. Macq B. Olivier E. Thiran J. (2006). “Normalization of transcranial magnetic stimulation points by means of Atlas registration,” in European Signal Processing Conference EUSIPCO2006: Vol. 14. 14th European Signal Processing Conference EUSIPCO2006 (Florence, Italy), 1–5.

